# The significance of the extent of tissue embedding for the detection of incidental prostate carcinoma on transurethral prostate resection material: the more, the better?

**DOI:** 10.1007/s00428-022-03331-6

**Published:** 2022-06-17

**Authors:** Jens Köllermann, Benedikt Hoeh, Daniel Ruppel, Kevin Smith, Henning Reis, Mike Wenzel, Felix Preisser, Marina Kosiba, Philipp Mandel, Pierre I. Karakiewicz, Andreas Becker, Felix K. H. Chun, Peter Wild, Luis A. Kluth

**Affiliations:** 1 Dr. Senckenberg Institute of Pathology, University Hospital Frankfurt, Goethe University Frankfurt am Main, Theodor-Stern-Kai 7, 60590 Frankfurt am Main, Germany; 2 Department of Urology, University Hospital Frankfurt, Goethe University Frankfurt am Main, Frankfurt/Main, Germany; 3 Cancer Prognostics and Health Outcomes Unit, Division of Urology, University of Montréal Health Center, Montreal, QC Canada

**Keywords:** Holmium laser enucleation, HoLEP, Transurethral resection of the prostate, TUR-P, Incidental prostate cancer, Prostate cancer

## Abstract

The aim of this study is to investigate the incidental prostate cancer (iPCa) detection rates of different embedding methods in a large, contemporary cohort of patients with bladder outlet obstruction (BOO) treated with transurethral surgery. We relied on an institutional tertiary-care database to identify BOO patients who underwent either transurethral loop resection or laser (Holmium:yttrium–aluminium garnet) enucleation of the prostate (HoLEP) between 01/2012 and 12/2019. Embedding methods differed with regard to the extent of the additional prostate tissue submitted following the first ten cassettes of primary embedding (cohort A: one [additional] cassette/10 g residual tissue vs. cohort B: complete embedding of the residual tissue). Detection rates of iPCa among the different embedding methods were compared. Subsequently, subgroup analyses by embedding protocol were repeated in HoLEP-treated patients only. In the overall cohort, the iPCa detection rate was 11% (46/420). In cohort A (*n* = 299), tissue embedding resulted in a median of 8 cassettes/patient (range 1–38) vs. a median of 15 (range 2–74) in cohort B (*n* = 121) (*p* < .001). The iPCa detection rate was 8% (23/299) and 19% (23/121) in cohort A vs. cohort B, respectively (*p* < .001). Virtual reduction of the number of tissue cassettes to ten cassettes resulted in a iPCa detection rate of 96% in both cohorts, missing one stage T1a/ISUP grade 1 carcinoma. Increasing the number of cassettes by two and eight cassettes, respectively, resulted in a detection rate of 100% in both cohorts without revealing high-grade carcinomas. Subgroup analyses in HoLEP patients confirmed these findings, demonstrated by a 100 vs. 96% iPCa detection rate following examination of the first ten cassettes, missing one case of T1a/ISUP 1. Examination of 8 additional cassettes resulted in a 100% detection rate. The extent of embedding of material obtained from transurethral prostate resection correlates with the iPCa detection rate. However, the submission of 10 cassettes appears to be a reasonable threshold to reduce resource utilization while maintaining secure cancer detection.

**Supplementary Information:**

The online version contains supplementary material available at 10.1007/s00428-022-03331-6.

## Introduction

Bladder outlet obstruction (BOO) and resultant lower urinary tract symptoms due to benign prostatic hyperplasia represent a frequent condition in men and its prevalence is increasing with age [1–3]. For the past few decades, transurethral resection of the prostate (TUR-P) has been the gold standard in the surgical treatment of LUTS/BOO management for low and medium-sized prostate volumes, after the failure of pharmacology treatment. [4–6]. However, several other treatment technologies have been introduced and validated in recent years [6–8]. Among those, Holmium:yttrium–aluminium garnet laser enucleation of the prostate (HoLEP) has emerged its way into current international guidelines as an alternative to TUR-P, demonstrating comparable efficacy and more favorable safety/tolerability [3, 4, 9, 10].

Regardless of the technique used, surgically obtained prostate tissue is routinely submitted for histological examination because, despite its assumed benign nature, clinically relevant iPCa requiring further diagnosis and therapy may occasionally be found [11, 12]. To maintain a rational balance between workload and diagnostic accuracy, guidelines are available that provide the pathologist with recommendations regarding the amount of tissue to be examined per patient [11, 12]. However, these are mainly based on TUR-P-only case series [13, 14]. Compared to standard TUR-P, considerably more prostate tissue is removed by HoLEP [11]. Consequently, more prostate tissue has to be submitted available for histological examination, resulting in a relevant increase in workload and costs. As of the time of writing, no validation of these guidelines has been performed in a contemporary cohort. We hypothesized that the current recommendations for histological evaluation may no longer represent the optimal balance between diagnostic safety and economic viability. To address this issue, we investigated the histologic carcinoma detection rates of two different tissue embedding methods on a large contemporary cohort of BOO patients who received transurethral treatment.

## Material and methods

### Study population

Within our prospectively-maintained institutional database, patients treated with endoscopic surgery (TUR-P, HoLEP) for BOO were retrospectively selected between 01/2012 and 12/2019. Patients undergoing palliative surgical treatment (histologically confirmed prostate cancer prior to treatment) were excluded from further analyses.

Transrectal/transabdominal ultrasound volumetry was performed to calculate preoperative prostate gland volume at the time of presentation. Patients’ characteristics were ascertained by a review of the medical chart. Preoperative assessments for prostate cancer detection were performed in line with current guidelines and decision was made in a shared-decision manner [4, 11, 15].

The current study was approved by the local institutional review boards of the University Cancer Centre and the local Ethical Committee (SUG-6–2018, 4/09) and is in line with the 1964 Helsinki declaration and its later amendments or comparable ethical standards [16].

### Tissue processing and embedding

Prostate tissue was fixated with 10% neutral buffered formalin and embedded in standard tissue cassettes, as described previously [17]. Between 01/2012 and 03/2019, tissue embedding was performed according to the current german (S3) guidelines, with slight modification [11]. Irrespectively of the total weight of the prostate tissue, up to ten cassettes were filled with prostatic tissue depending on the amount of resected tissue. In the case of residual tissue, another cassette was prepared for every 10 g of residual tissue. This procedure represents our protocol for standardized selective tissue embedding, and this protocol was used in cohort A. By contrast, between 04/2019 and 12/2019, all prostate tissue was embedded, irrespective of the sample weight (cohort B).

### Histopathological examination

Histopathological examination was based on standardized 3 µm hematoxylin–eosin-stained slides and was performed by board-certified pathologists. The order in which the slides were reviewed paralleled that in which the corresponding tissue was embedded. Additional immunohistochemical staining (antibodies: alpha-methylacyl-CoA racemase, AMACR, clone 13H4, GA060; Agilent, Santa Clara, CA, USA, and high molecular weight keratin clone 34betaE12, GA051, Agilent, Santa Clara, CA, USA) was performed to either clarify findings suspicious for carcinoma or to confirm a diagnosis of carcinoma. Histopathological reporting was performed according to the TNM-classification (8^th^ edition) as well as to the recommendations of the 2019 International Society of Urological Pathology (ISUP) Consensus Conference on Grading of Prostatic Carcinoma [18–20]. Histopathological examination over the above-mentioned periods was performed by a total of 11 board-certified pathologists, including two uropathologists.

To evaluate the significance of the extent of tissue embedding on histologic carcinoma detection and to determine an upper cutoff, all histologic slides from all patients with an initial diagnosis of incidental prostate cancer were reviewed by a uropathologist (JK). The slides were examined in the order in which the corresponding tissue was embedded, and the slide number of each tumor-bearing section was noted. Example: patient xy: slide number 2, 4, and 7 out of 10 slides showed tumor.

Thus, the minimum number of slides (= number of tissue blocks) required for the diagnosis of prostatic carcinoma was noted in each case (virtual downsampling).

### Statistical analyses

Descriptive statistics included frequencies and proportions for categorical variables. Means and medians (minimum–maximum) were reported for continuously coded variables. The chi-square test and Fisher’s exact test were used for statistical significance in proportions’ differences. The Wilcoxon-Mann–Whitney *U* test examined the statistical significance of means and distribution differences. Sensitivity for prostate cancer detection was calculated following histological examination of the first ten cassettes. Given the clinical context, specificity was considered to be 100%. First, separate sensitivity rates were calculated for the different embedding protocols (cohort A vs. cohort B), regardless of surgical approach (TUR-P vs. HoLEP). To detect possible influence (bias) of the unequal distribution of surgical methods in both cohorts on the cancer detection rate, a subgroup analysis was performed including only patients treated with HoLEP. All tests were two sided with a level of significance set at *p* < 0.05. The “BiAS” environment for statistical computing and graphics was used for all analyses.

## Results

### Descriptive characteristics of overall cohort

Relying on our institutional database, we identified 420 eligible patients, who underwent endoscopic surgical treatment for BOO at the University Hospital Frankfurt between 01/2012 and 12/2019. Of these, 71% (299/420) prostate tissue samples were examined using a guideline-derived (limited) embedding protocol (cohort A), which resulted in complete tissue embedding in 60% (177/299) of these samples. In cohort B (29%; 121/420), a “complete submission” prostate tissue embedding protocol was used, irrespectively of tumor weight.

The patients were divided into two cohorts. In cohort A (*n* = 299), the prostate tissue samples were examined using a guideline-derived embedding protocol, which resulted in complete tissue embedding in 60% (177/299) of these cases. In cohort B (*n* = 121), a “complete submission” prostate tissue embedding protocol, irrespectively of tumor weight.

In cohort A, HoLEP was performed in 53% (158/299) patients versus 98% (119/121) in cohort B (*p* < 0.001). Cohort A differed from cohort B with regards to lower median preoperative prostate volume (60 vs. 68.5 cm^3^; *p* = 0.013), lower medium tissue weight (27 vs. 45 g; *p* < 0.001), and lower preoperative proportions of PSA ≥ 20 ng/ml (1.8 vs. 2.5%; *p* = 0.043). By contrast, no statistically significant differences were found for PSA-density, age, or preoperative prostate biopsy frequencies (both *p* > 0.3). Patients’ characteristics are summarized in Table 1.

**Table 1:** Overall cohort (*n* = 420): Comparison of patient characteristics between cohort A (guideline-adapted tissue embedding) and cohort B (complete tissue embedding); all values are frequencies (%), means, or medians (minimum–maximum)

	Cohort A (*n* = 299, 71.2%)	Cohort B (*n* = 121, 28.8%)	*p* value
Age in [years], Median (Min.-Max.)	71 (39–98)	70 (49–91)	0.36
Mean	69.6	69.0	
Prostate volume in [cm^3^]	*n* = 271	*n* = 116	
Median (Min.-Max.)	60 (12–230)	68.5 (24–210)	0.013
Mean	68.5	76.2	
Tissue weight in [g]	*n* = 299	*n* = 121	
Median (Min.-Max.)	27 (1–206)	45 (5–232)	< 0.001
Mean	41	54.2	
PSA in [ng/ml], *n* (%)	*n* = 278	*n* = 119	
≤ 10	246 (88.5%)	94 (79.0%)	0.043
> 10– < 20	27 (9.7%)	22 (18.5%)	
≥ 20	5 (1.8%)	3 (2.5%)	
PSA/volume–ratio in [ng/ml*cm^3^], *n* (%)	*n* = 197	*n* = 103	
< 15	27 (13.7%)	21 (20.4%)	0.322
15–20	49 (24.9%)	23 (22.3%)	
> 20	121 (61.4%)	59 (57.3%)	
Prostate biopsy, *n* (%)	34 (11.4%)	13 (10.7%)	0.989

*PSA* prostate-specific antigen, *Min*. minimum, *Max*. maximum

With respect to the surgical procedures used, significantly more tissue was obtained with the HoLEP procedure compared to the conventional TUR-P procedure (median 49 g (95% CI: 53.1–63.3) vs. 15.0 g (95% CI: 15.6–20.35); *p* < 0.0001).

Average tissue weight per cassette for TUR-P specimen was 1.70 g vs. 3.03 g for HoLEP, respectively.

### Clinicopathological characteristics of overall cohort

In the overall cohort, iPCa was detected in 11% (46/420) of patients. Of these, 8% (23/290) were recorded in cohort A and 19% (23/121) cases in cohort B (*p* < 0.001). ISUP-Grading and T-stage distributions were comparable between both cohorts with predominantly ISUP-Grade I/II (91 vs. 100%; *p* = 0.46) and stage T1a (70 vs. 91%; *p* = 0.14) features found in cohort A vs. cohort B, respectively. The number of cassettes per case (cassettes/case-ratio) was significantly lower in cohort A compared to cohort B (8 vs. 15; *p* < 0.001). Clinicopathological characteristics are summarized in Table 2.

**Table 2:** Overall cohort (*n* = 420): Comparison of clinicopathological characteristics between cohort A (guideline-adapted tissue embedding) and cohort B (complete tissue embedding); all values are frequencies (%), means, or medians (minimum–maximum)

	Cohort A (*n* = 299, 71.2%)	Cohort B (*n* = 121, 28.8%)	*p* value
Incidental prostate cancer, *n* (%)	23 (7.7%)	23 (19.0%)	0.001
Surgical approach, *n* (%)			
HoLEP	158 (52.8%)	119 (98.3%)	< 0.001
TUR-P	141 (47.2%)	2 (1.7%)	
ISUP-Grading, *n* (%)			
1	14 (60.9%)	17 (73.9%)	0.46
2	7 (30.4%)	6 (26.1%)	
≥ 3	2 (8.7%)	0 (0%)	
T-stage, *n* (%)			
T1a	16 (69.6%)	21 (91.0%)	0.14
T1b	7 (30.4%)	2 (9.0%)	
Total number of cassettes, *n*	1413	2124	
Cassettes/case-ratio, Median (Min.-Max.)	8 (1–38)	15 (2–74)	< 0.001
Mean	9.6	17.7	

*HoLEP* (HO:YAG) laser enucleation of the prostate, *TUR*-*P* transurethral resection of the prostate, *ISUP* International Society of Urological Pathology, *Min*. minimum, *Max*. maximum, *n*.*a*. not applicable

### Carcinoma detection rate per number of cassettes examined (overall cohort)

**Fig. 1:**
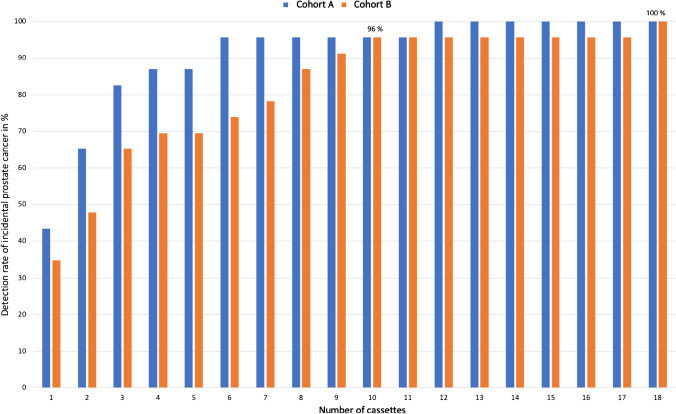
Overall cohort (*n* = 420): Bar plot depicting the detection rate of incidental prostate cancer in relation to the number of cassettes, examined histologically and stratified by type of tissue embedding protocol (cohort A vs. cohort B)

Virtual reduction of the number of cassettes to ten resulted in a carcinoma detection rate of 96% in cohort A and cohort B. Increasing the number of cassettes by two and eight cassettes, respectively, resulted in a detection rate of 100% in both cohorts. Of note, pathological findings derived from additional review (i.e., after the initial diagnosis of prostate cancer was determined), did not lead to changes in ISUP grade or T-stage in any of the cases (Fig. 1).

### Descriptive characteristics of the HoLEP cohort

The HoLEP-only subgroup included 277 patients in total. In 158 of these patients (57%), embedding of the resected tissue was performed according to the modified guideline recommendation (cohort A). In contrast, the tissue samples of the remaining 119 patients (cohort B) were fully embedded. No significant differences in patient characteristics were found (all *p* ≥ 0.2, Table 3).

**Table 3:** HoLEP cohort (*n* = 277): Comparison of patient characteristics between cohort A (guideline-adapted tissue embedding) and cohort B (complete tissue embedding); all values are frequencies (%), means, or medians (minimum–maximum)

	Cohort A (*n* = 158, 57.0%)	Cohort B (*n* = 119, 43.0%)	*p* value
Age in [years], Median (Min.-Max.)	70 (45–98)	70 (49–91)	0.51
Mean	69.8	69.2	
Prostate volume in [cm^3^]	*n* = 151	*n* = 115	
Median (Min.-Max.)	73 (12–230)	69.0 (24–210)	0.24
Mean	82.9	76.6	
Tissue weight in [g]	*n* = 158	*n* = 119	
Median (Min.-Max.)	52 (1.5–206)	45 (5–232)	0.23
Mean	61.3	54.9	
PSA in [ng/ml], *n* (%)	*n* = 148	*n* = 117	
≤ 10	122 (82.4%)	92 (78.6%)	0.68
> 10– < 20	22 (14.9%)	22 (18.8%)	
≥ 20	4 (2.7%)	3 (2.6%)	
PSA/volume–ratio in [ng/(ml*cm^3^], *n* (%)	*n* = 93	*n* = 101	
< 15	15 (16.2%)	21 (20.8%)	0.44
15–20	27 (29.0%)	22 (21.8%)	
> 20	51 (54.8%)	58 (57.4%)	
Prostate biopsy, *n* (%)	13 (8.2%)	13 (10.9%)	0.58

*PSA* prostate-specific antigen, *Min*. minimum, *Max*. maximum

### Clinicopathological characteristics of the HoLEP cohort

In patients treated with HoLEP (*n* = 277), the iPCa rate was 12% (*n* = 32). Of these, 5% (*n* = 9) were recorded in cohort A whereas 19% (*n* = 23) in cohort B (Table 4; *p* < 0.001). ISUP-Grading and T-stage distributions were comparable among both cohorts, with predominantly ISUP-Grade I/II (89 vs. 100%; *p* = 0.13) and T1a (89 vs. 91%; *p* = 1.00) features in cohort A vs. B, respectively. The cassette/case-ratio was significantly lower in cohort A vs. B (8 vs. 15; *p* < 0.001). Conversely, the percentage of tumor-bearing cassettes relative to the total number of cassettes per case (tumor cassette/total cassette ratio) was significantly lower in cohort B (*p* = 0.017). Clinicopathological characteristics are summarized in Table 4.

**Table 4:** HoLEP cohort (*n* = 277): Comparison of clinicopathological characteristics between cohort A (guideline-adapted tissue embedding) and cohort B (complete tissue embedding); all values are frequencies (%), means, or medians (minimum–maximum)

	Cohort A (*n* = 158, 57.0%)	Cohort B (*n* = 119, 43.0%)	*p* value
Incidental prostate cancer, *n* (%)	9 (5.7%)	23 (19.3%)	< 0.001
ISUP-Grading, *n* (%)			
1	4 (44.4%)	17 (73.9%)	0.13
2	4 (44.4%)	6 (26.1%)	
≥ 3	1 (11.2%)	0 (0%)	
T-stage, *n* (%)			
T1a	8 (88.9%)	21 (91.3%)	1.00
T1b	1 (11.1%)	2 (8.7%)	
Total number of cassettes, *n* (%)	1413 (100%)	2124 (100%)	
Cassettes/case ratio, Median (Min.-Max.)	8 (1–33)	15 (2–74)	< 0.001
Mean	8.9	17.9	

*ISUP* International Society of Urological Pathology, *Min*. minimum, *Max*. maximum

### Cancer detection rate according to the number of examined cassettes (HoLEP cohort)

Virtual reduction of the number of cassettes to ten cassettes resulted in a carcinoma detection rate of 100 vs. 96% for cohort A vs. cohort B, respectively.

**Fig. 2:**
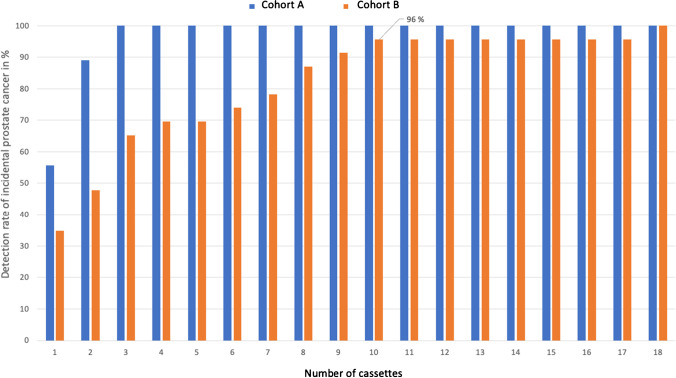
HoLEP cohort (*n* = 277): Bar plot depicting the detection rate of incidental prostate cancer in relation to the number of cassettes, examined histologically and stratified by type of tissue embedding protocol (cohort A vs. cohort B)

Increasing the number of cassettes by eight cassettes in cohort B resulted in a detection rate of 100% and revealed one further case of a stage T1a/ISUP grade 1 prostate cancer diagnosis. Of note, pathological findings derived from additional review (i.e., after initial diagnosis of prostate cancer was determined), did not lead to changes in ISUP grade or T-stage in any of the cases (Fig. 2).

## Discussion

Histologic examination of prostate tissue obtained by transurethral surgery from BOO-patients is performed to exclude iPCa. The current literature shows detection rates ranging from 1 to 23% (Supplementary Table 1) [5, 13, 21–34]. In view of the frequency of the disease and the highly variable incidence rates, the extent of tissue embedding is of great importance; however, diagnostic safety (i.e., probability of detecting carcinoma) must be balanced by the practical limits imposed by economics. In this regard, our investigation revealed several noteworthy findings.

### Increased carcinoma detection rate following extended tissue examination

The overall cohort studied showed an incidence rate of 11%; the cohort with complete tissue embedding showed a rate of 19%. Both rates are in the range of values reported in the literature (see Supplementary Table 1). Interestingly, the comparison of the incidence rates between the cohort with limited (cohort A) vs. complete tissue embedding (cohort B) showed a significantly higher carcinoma detection rate for the latter cohort (8 vs. 19%; *p* = 0.001). The assumption of a causal relationship between the amount of tissue examined and the carcinoma detection rate is therefore reasonable. However, other causes for this finding must also be considered.

### Dependence on surgical method and patient selection

Significant differences in the frequency of surgical techniques used (TUR-P vs. HoLEP) were noted between cohorts A and B. The proportion of HoLEP in cohort A was 53 vs. 98% in cohort B, raising the question of whether these differences may have influenced the iPCa rate.

A comparative study on iPCa rates after conventional loop technique versus HoLEP procedure was presented by Herlemann et al. [21]. The authors stated that surgical technique had no significant effect on the detection rate of iPCa (15% for TUR-P (39/229) and 17% for HoLEP (43/289); *p* = 0.593). It is of note that the patient cohort in Herlemann et al.’s study differed substantially with respect to clinical and histopathological relevant parameters, such as patient age (*p* = 0.007), absolute PSA level (*p* < 0.001), prostate volume (*p* < 0.001), PSA density (*p* = 0.001), presence of preoperative core biopsy (*p* < 0.001), resected weight (*p* < 0.001), and relative resected tissue percentage based on prostate volume (*p* < 0.001). However, these parameters should be considered relevant factors with regard to the detection of iPCa [21, 32–34]. The validity of this study should be critically evaluated under this light. In contrast to the above study, Rosenhammer et al. addressed the same question in a matched-pairs analysis and found a significantly higher iPCa rate in patients submitted to the HoLEP laser procedure (8 vs. 23%) [23]. The most plausible explanation given by the authors was a more extensive tissue removal. This would particularly affect the peripheral zone, as the site of origin of most prostate carcinomas. While we were able to confirm increased tissue ablation using the HoLEP procedure compared to the classic loop procedure, we were unable to find confirmation of increased resection of peripheral zone tissue in the literature. This hypothesis also contradicts our own clinical experience (2021: *n* > 280 cases), according to which precise enucleation of the adenomatous transitional zone is usually possible by dissection along the anatomic tissue plane between the peripheral and transitional zone. In view of the contradictory data in the literature, we tried to exclude the type of surgery as a causative factor for the increased carcinoma detection rate, relying on subgroup analyses, which only included patients receiving the HoLEP procedure. With a comparable risk profile of these patients, analyses persistently showed a significantly higher carcinoma detection rate for the cohort with complete tissue embedding (19 vs. 6%, *p* < 0.001). Thus, it remains reasonable to assume that examination of a larger volume of tissue is associated with higher carcinoma detection rate rather than the choice of the operative procedure. However, this led to significantly increased tissue embedding (up to 74 cassettes /case) and consequently, to a significant additional workload. Therefore, the question arises whether this additional effort is necessary from a diagnostic point of view, and thus required for routine diagnostics.

### Diagnostic benefit of extended tissue embedding is limited by an upper cutoff

We addressed this question by virtual down-sampling of the tissue blocks. After taking just the first ten blocks/patient into account, a sensitivity of 100% (cohort A) and 96% (cohort B) of carcinoma was recorded and no tumor with Gleason pattern > 3 or stage > T1a was missed. After examination of a maximum of 18 cassettes, a detection rate of 100% was also achieved in cohort B. This indicates that the diagnostic benefit of increased tissue embedding is only present up to a certain cut-off. Tissue embedding beyond this cutoff therefore appears to be of no further diagnostic benefit. Similar results were also recorded by Murphy et al. [24]. The authors compared two cohorts in which an incomplete (examination of up to 12 g of resected tissue) and a complete tissue examination after TUR-P was performed. No significant difference between the cohorts was found in terms of carcinoma detection rate. Thus, examination of 12 g of prostatic tissue allowed detection of 90% of all iPCa and 100% of all clinically significant neoplasms.

However, the study by Newman et al. led to the opposite result [13]. Here, two cohorts with 500 cases each were compared after classical TUR-P. In the first cohort, tissue embedding was complete, and in the second cohort incomplete. Significantly more iPCa, including clinically significant carcinomas, were found after complete tissue embedding (14 (*n* = 71/500) vs. 9% (*n* = 43/500); *p* < 0.01). Therefore, the authors recommended complete tissue embedding as a standard [13]. Unfortunately, the authors did not address the question of a possible upper limit for tissue embedding in their study, which limits the value of the study’s conclusion and therefore does not invalidate our results.

### Impact on guideline recommendations on tissue embedding

Our results appear to be of great practical relevance considering the current national guideline recommendations on tissue embedding. With the aim of achieving a reasonable balance between workload and diagnostic safety, the current German recommendation is as follows: “[…] *transurethral prostate resection material should be weighed and subsequently at least 10 cassettes should be embedded. Of the remaining material, one additional capsule should be embedded for every 3 g*” [11]. The key issue for limited tissue embedding in this recommendation is knowing the amount of tissue a cassette can be loaded with (defined as cassette fill weight). If the capacity per cassette is less than 3 g of tissue, subtotal embedding of the residual tissue will be the result. However, if the capacity is 3 g or more, complete tissue embedding is inevitable. For this purpose, we made calculations using our own material. The 298 cases in which the entire tissue was embedded were used. By dividing the measured total tissue weight by the number of tissue cassettes prepared, the tissue holding capacity per capsule could be calculated. It amounted to a mean of 2.4 g (median 2.1 g) per capsule. A subdivision depending on the surgical method used (TUR-P vs. HoLEP) revealed differences regarding the capacity per capsule. For tissue obtained by TUR-P, the tissue capacity per capsule was 1.70 g on average, whereas by HoLEP technique, the capacity per capsule was 3.03 g on average.

This difference can be explained by technical factors. In the HoLEP procedure, tissue fragments are laser-resected which, due to their large size, require secondary intravesical mechanical morcellation. The resulting tissue fragments are therefore significantly smaller than the fragments obtained after TUR-P, which do not need secondary morcellation. In consequence, the cassettes can be loaded more densely and hence, with more tissue.

In summary, these observations and calculations demonstrate that with respect to the HoLEP procedure, the implementation of the guideline specification for tissue embedding does not correspond to tissue sampling but results in complete embedding. If the cutoff of 10 cassettes was used for the HoLEP material instead of the guideline recommendation, a significant reduction in the number of cassettes could be achieved. Relying on the current data, this modification would result in a reduction by 43% (1210 instead of 2124 cassettes). Considering the high frequency of this disease and thus the frequent need for surgical intervention, the expected savings potential would be of considerable health economic relevance [35–37].

In addition to the economic reasons, a restriction of tissue embedding also appears reasonable from a medical point of view. The aim of histological examination should be to exclude the presence of a high-grade carcinoma requiring treatment. This appears to be the case using the cut-off we established in our study. Tissue embedding that goes beyond this leads to a further (albeit slight) increase in the carcinoma detection rate, but generally detects only insignificant carcinomas that do not require further treatment.

Despite noteworthy findings, our study is not devoid of limitations. We acknowledge the retrospective design of the study. Furthermore, the absolute number of detected carcinomas in our study is relatively small. The conclusions we have drawn therefore require confirmation by further studies, preferably with even larger numbers of cases.

Moreover, results should be interpreted in the light of a single-institution cohort. Furthermore, histopathological examination was performed by a total of eleven different pathologists. Therefore, a certain degree of interobserver variance can be assumed. Although its influence on the carcinoma detection rate remains unclear, the scenario represents the real-world situation in clinical practice very well.

## Conclusion

The extent of embedding of material obtained from transurethral prostate resection correlates with the prostate cancer detection rate. Our study shows that the correlation is not linear, but rather shows an upper threshold of 10 tissue cassettes above which further tissue embedding brings no additional diagnostic benefit. Thus, a significant reduction of tissue embedding with consequently reduced workload and reduced cost is possible. Therefore, the existing guidelines on the extent of tissue embedding should be critically revised, particularly with regard to the HoLEP procedure.

## Supplementary Information

Below is the link to the electronic supplementary material.

Supplementary file1 (DOCX 18.3 KB)
