# Supplementary material for: The significance of the extent of tissue embedding for the detection of incidental prostate carcinoma on transurethral prostate resection material: the more, the better?
Source: Virchows Arch. 2022 Jun 17;481(3):387–96. doi: 10.1007/s00428-022-03331-6 (PMC9485088; doi:10.1007/s00428-022-03331-6)
Supplement: Supplementary file 1 — Supplementary file1 (DOCX 18.3 KB) [file 428_2022_3331_MOESM1_ESM.docx]

|  | Cohort A  (n = 299, 71.2%) | Cohort B  (n = 121, 28.8%) | p-value |
| --- | --- | --- | --- |
| Age in [yrs],  Median (Min.-Max.) | 71  (39 - 98) | 70  (49 - 91) | 0.36 |
| Mean | 69.6 | 69.0 |  |
| Prostate volume in [cm^3^] | n=271 | n=116 |  |
| Median (Min.-Max.) | 60  (12 - 230) | 68.5  (24 - 210) | 0.013 |
| Mean | 68.5 | 76.2 |  |
| Tissue weight in [g] | n=299 | n=121 |  |
| Median (Min.-Max.) | 27 (1-206) | 45 (5-232) | <0.001 |
| Mean | 41 | 54.2 |  |
| PSA in [ng/ml],  n (%) | n = 278 | n = 119 |  |
| ≤ 10 | 246 (88.5%) | 94 (79.0%) | 0.043 |
| > 10 - < 20 | 27 (9.7%) | 22 (18.5%) |  |
| ≥ 20 | 5 (1.8%) | 3 (2.5%) |  |
| PSA/volume–ratio  in [ng/ml*cm^3^],  n (%) | n = 197 | n = 103 |  |
| < 15 | 27 (13.7%) | 21 (20.4%) | 0.322 |
| 15 – 20 | 49 (24.9%) | 23 (22.3%) |  |
| > 20 | 121 (61.4%) | 59 (57.3%) |  |
| Prostate biopsy,  n (%) | 34 (11.4%) | 13 (10.7%) | 0.989 |

Table 1 Summary of studies investigating incidental prostate cancer (iPCa) rates in transurethral resection (TUR-P) or laser (Holmium:yttrium-aluminium garnet) enucleation of the prostate (HoLEP) treated patients for bladder outlet obstruction

Abbreviations: PSA= Prostate-specific antigen; Min.=Minimum; Max.=Maximum
